# Osteoporosis in Rheumatic Diseases

**DOI:** 10.3390/ijms20235867

**Published:** 2019-11-22

**Authors:** Giovanni Adami, Angelo Fassio, Maurizio Rossini, Cristian Caimmi, Alessandro Giollo, Giovanni Orsolini, Ombretta Viapiana, Davide Gatti

**Affiliations:** Rheumatology Unit, University of Verona, Policlinico Borgo Roma, Pz Scuro 10, 37134 Verona, Italy; angelo.fassio@yahoo.it (A.F.); maurizio.rossini@univr.it (M.R.); cristian.caimmi@gmail.com (C.C.); alessandrogiollo@gmail.com (A.G.); giovanniorsolini@gmail.com (G.O.); ombretta.viapiana@univr.it (O.V.); davide.gatti@univr.it (D.G.)

**Keywords:** osteoporosis, rheumatology, rheumatic diseases, inflammation, arthritis

## Abstract

Osteoporosis is a chronic disease characterized by an increased risk of fragility fracture. Patients affected by rheumatic diseases are at greater risk of developing osteoporosis. The purpose of the present review is to discuss the pathogenesis, epidemiology, and treatment of osteoporosis in patients affected by rheumatic diseases with special focus for rheumatoid arthritis, psoriatic arthritis, spondyloarthritis, systemic lupus erythematosus, systemic sclerosis, vasculitides, Sjogren syndrome, and crystal-induced arthritis.

## 1. Introduction

Rheumatic diseases are characterized by predominant inflammation (autoimmune arthritides) and/or tissue deterioration (e.g., systemic sclerosis). Osteoporosis (OP) is a hallmark of rheumatic diseases, and its prevalence is destined to grow in the next years given the ageing of rheumatic patients [1]. Inflammation and immobility are among the principal pathways leading to bone loss in rheumatic diseases, but other metabolic mechanisms are involved in OP pathogenesis. These mechanisms have mostly been studied in postmenopausal OP and other rare monogenic skeletal diseases, but similar considerations can be made when dealing with inflammatory conditions. In particular, the receptor activator of the nuclear factor kappa-Β ligand (RANKL)/osteoprotegerin (OPG) and Wnt pathways are the principal regulators of bone remodeling [2]. RANKL is a cytokine of the tumor necrosis factor (TNF) family and, with its decoy molecule OPG, regulates the activity of osteoclasts. RANKL has been shown to be essential for osteoclast maturation and development [3]. In contrast, the Wnt/β-catenin pathway regulates osteoblast differentiation by activating the transcription of osteoblast-specific genes and acting as a major regulator of osteogenesis [2]. Wnt inhibitors, Dickkopf-related protein 1 (Dkk-1), and sclerostin counteract the activity of the Wnt system by bonding with the Wnt transmembrane receptors, LRPs and Frizzled. In addition, Dkk-1 and sclerostin increases have been associated with the activation of osteoclasts. In rheumatic diseases, with specific exceptions that will be discussed later in the review, i.e., Wnt inhibitor and RANKL secretion, are intensified, resulting in deleterious effects for bone. Fortunately, clinicians can employ several antiosteoporotic medications that can effectively prevent OP fractures from occurring. OP drugs can be divided into antiresorptive agents (e.g., bisphosphonates and denosumab) and bone anabolic agents (e.g., teriparatide and abaloparatide). Romosozumab, a monoclonal antibody used against sclerostin, is a novel and recently-approved molecule which acts upon both bone resorption and bone formation [4]. Anti-resorptive agents reduce the risk of fracture by inhibiting the activity of osteoclasts. Bisphosphonates can bind hydroxyapatite crystals and, when incorporated into the cytoplasm, lead to the death of the osteoclast by inhibiting enzymes in the mevalonate pathway [5]. Denosumab, a RANKL inhibitor, is a potent inhibitor of bone resorption but, in contrast to bisphosphonates that can reside into the bone for years, has an on/off mechanism of action [6]. Teriparatide and abaloparatide are analogs of the parathyroid hormone (PTH) whose intermittent use leads to osteoblast activation, and eventually, bone matrix deposition [7]. In the present review, the pathophysiology of osteoporosis and its treatment in the context of rheumatic diseases is discussed.

## 2. Rheumatoid Arthritis

Local and systemic bone loss are hallmarks of rheumatoid arthritis (RA) that result from the deterioration of both trabecular and cortical bone [8,9]. The pathogenesis of bone loss at local and systemic levels predominantly involves inflammatory status, the release of cytokine and the production of autoantibodies. Systemic osteopenia occurs in the early stages of RA and, according to a recently-published study, even before the onset the disease [10]. In RA-related osteoporosis (OP), the whole bone is affected, although cortical sites (i.e., femoral neck and distal radius) seem to be more susceptible to bone loss [11]. Indeed, high-resolution peripheral quantitative computed tomography (HRpQCT) has indicated that RA patients have increased cortical porosity [12,13] with reduced mechanical strength [14], which results in a greater risk of fragility fractures compared with healthy controls [14]. Inflammation in RA is mainly driven by augmented cytokine secretion, including TNF-α, Interleukin-6 (IL-6), and Interleukin-1 (IL-6). These cytokines can directly and indirectly activate osteoclasts, inducing bone loss. Moreover, inflammatory cytokines can halt osteoblast differentiation. In addition, inflammation can lead to osteoporosis through the systemic and local release of proteinases (metalloproteinases) that can directly degrade bone tissue. RANKL is one of the key cytokines involved in the pathogenesis of local and systemic bone loss in RA. In post-menopausal women with OP, the surface RANKL is expressed by osteoblasts and enhances osteoclast activity [15]. In contrast, in RA patients, the principal source of RANKL is CD4+CD28- T cells, and in this setting, RANKL was shown to exert both a positive effect on osteoclastogenesis and detrimental effect on the development of osteoblasts [16,17]. Dkk-1, a Wnt signaling inhibitor, is another major regulator of joint remodeling [18] whose increase was associated with greater risk of OP and bone erosion in RA patients [19]. According to a recent meta-analysis, Dkk-1 serum levels were significantly higher in RA compared to controls [20]. Dkk-1 production is enhanced mainly by inflammatory cytokines, such as TNF [18], but emerging evidence has shown that PTH can mediate part of the secretion of Dkk-1 [21,22,23], especially in RA [19]. The PTH-driven increase of Dkk-1 might explain, at least in part, the unfavorable outcome of RA patients with low-vitamin D status [24,25,26]. Other independent factors associated with bone destruction in RA are autoantibodies against citrullinated proteins (ACPA) [27,28]. ACPA positivity is related, in a titer-dependent manner, to systemic OP [29,30], even before the clinical onset of RA [10]. In addition, ACPA-positive RA patients showed precocious metacarpal periarticular osteopenia [31] and, interestingly, ACPA-positive psoriatic arthritis (PsA) patients occasionally displayed a comparable periarticular bone loss that was not demonstrated in ACPA-negative PsA patients [32]. All this evidence supports the hypothesis of the direct activation of osteoclasts by ACPA [27].

OP can be found in 30 to 50% of RA patients [33,34,35], and the risk of developing OP in RA was strongly associated with the duration and severity of the disease, as well as with the age and sex of the patients [11,36]. Therefore, the risk of OP is higher in RA pre-menopausal women compared to age-matched healthy controls [37]. Likewise, male individuals with RA have a two-fold increased risk of OP compared with controls [38]. In addition, two meta-analyses showed a 60 to 100% higher risk of fracture in RA patients compared to healthy controls [39,40]. Another meta-analysis, focused only on vertebral fractures, confirmed a similar two-fold increase in vertebral fracture risk in RA patients [41]. However, despite these alarming data, only about 45% of RA patients are receiving calcium and vitamin D supplements [42], and only 5.4% of RA patients who are not taking glucocorticoids (GCs) are using bisphosphonates [43]. In addition, among a cohort of 11,669 RA patients followed for a decade, only 35% were prescribed OP specific drugs, and only half of the patients at high risk of fracture received any OP medication [44].

GCs are widely used for the treatment of RA, and their efficacy in reducing inflammation is largely recognized [45]. Nonetheless, GCs have proven harmful effects on bone health; fractures are the most important adverse events related to their use [46,47,48,49,50,51]. Fracture incidence in newly-diagnosed RA patients exposed to GCs is twice as prevalent as it is among non-exposed patients [48]. Despite this evidence on long-term, high-dose treatment with GCs, much controversy remains on the true harmfulness of short-term and low-dose glucocorticoid course in active RA. These controversies are largely based on the established benefits of GCs in controlling disease activity [45]. In 1986, Sambrook and colleagues described a non-significant reduction of bone mineral density (BMD) in active RA patients treated with GCs compared with RA patients who did not received GCs, providing evidence on the safety of low-dose GCs in term of OP risk in RA [52]. Additionally, a more recent meta-analysis of randomized, placebo-controlled trials on the effect of GCs on BMD in active and early RA demonstrated that patients treated with GCs did not experience a significant BMD change compared to patients in the placebo group [53]. Another earlier meta-analysis showed that GCs were associated with a negative effect on lumbar spine but not hip BMD in RA patients [54]. Furthermore, the latter meta-analysis showed that RA patients treated with GCs did not lose hand BMD compared to patients not on GCs [54]. In conclusion, low-dose and short-term glucocorticoid treatment appears to be safe, at least in patients with early and active RA. Nevertheless, in 2016 a European League Against Rheumatisms (EULAR) task force recommended to systematically review, in all RA patient, the predisposing factors of OP, including glucocorticoid exposure, and, when applicable, to calculate 10-year risk of fracture with the fracture risk assessment algorithm (FRAX) [55]. However, the most diffuse version of the FRAX is not able to discriminate between long- and short-term users or between high and low doses of steroids [56], raising some concerns about the usefulness of FRAX in glucocorticoid-treated RA patients. For the latter reason, in many national guidelines on glucocorticoid-induced OP, FRAX is not included for the stratification of patient risk [57,58,59], and in others, is included only in the glucocorticoid-adjusted version [60,61].

Several studies have explored the efficacy of conventional disease modifying anti-rheumatic drugs (DMARDs) in the prevention of bone loss in RA patients, with controversial results [62]. Overall, studies have found a possible small positive effect on bone density and metabolism, probably mediated by the reduction of inflammation [63]. Some controversy also still remains regarding biological DMARDs and OP prevention in RA [64,65,66]. TNF inhibitors were the first molecules studied in this field, with conflicting results in BMD and bone turnover markers (BTMs) [64,65,67,68,69,70,71]. The variability of these results was probably related to the large number of confounding factors (such as vitamin D deficiency and corticosteroid therapy). A recently published study showed that TNF inhibitor use was associated with a significant, albeit small, vertebral fracture risk reduction in RA patients [72]. IL-6 inhibitors and abatacept showed encouraging results in preventing bone loss in RA, achieving better results on BMD than TNF inhibitors, especially at periarticular sites and in ACPA-positive patients [64,73,74]. Indeed, these promising results might be explained by the reduction of osteoclasts-activating antibodies [27]. However, a recently-published study on a small number of RA patients showed that 12 months’ treatment with Rituximab, a B-cell depleting treatment, induced a reduction in femoral neck BMD despite the improvement of disease activity [75]. The latter finding was particularly evident in vitamin-D-deficient patients, in line with the results of a previously-published study on TNF inhibitors [68].

Bisphosphonates are the most commonly prescribed medications for post-menopausal OP. Bisphosphonates have proven efficacy on fractures prevention [76], and have an acceptable safety profile [77]. The evidence of the efficacy of bisphosphonates in the treatment of OP in RA patients largely comes from the extrapolation of data from glucocorticoid-induced OP clinical trials [78,79,80,81]. RA represented the most frequent glucocorticoid requiring disease in these trials; the proportion of patients affected by RA ranged between 27 to 44% of the individuals enrolled [51]. Overall, these trials demonstrated the efficacy of bisphosphonates in terms of the prevention and treatment of glucocorticoid-induced OP in RA subpopulations. Moreover, zoledronic acid, a potent intravenous amino-bisphosphonate, appeared to offer protection from the development and progression of structural damage in RA when used together with methotrexate [82], and it has been recently hypothesized that it plays the role of amino-bisphosphonates in reducing circulating γδ T cells and controlling inflammation in RA [83]. Denosumab is a human monoclonal antibody against RANKL with a proven efficacy in preventing fractures in post-menopausal OP [84] and increasing BMD in glucocorticoid-induced OP [85]. Furthermore, denosumab has been shown to reduce cortical porosity and increase mechanical strength in both post-menopausal women and RA patients [86,87]. For the latter reason, there has been a growing interest regarding the use of denosumab in the treatment of RA [88]. In 2008, the Denosumab Rheumatoid Arthritis Study Group conducted a phase-II trial on the effects of denosumab on structural damage, BMD, and bone turnover in RA patients [89]. BMD increased from baseline by 3.0% at the lumbar spine and 1.6% at total hip (significantly more than the placebo). In the same trail, Denosumab, added to methotrexate, significantly inhibited bone erosion progression at 6 and 12 months of follow-up. In 2016, another clinical trial further validated the positive effect of denosumab in terms of bone erosion prevention in RA [90]. Moreover, in RA patients with OP, switching from oral bisphosphonates to denosumab resulted in a substantially lower progression of bone erosion compared to continuing bisphosphonates [91]. Interestingly, switching from bisphosphonates to teriparatide in RA resulted in the progression of bone erosion [91]. This outcome was somehow anticipated by a recently published placebo-controlled clinical trial on the efficacy of teriparatide in preventing bone erosions [92]. As a matter of fact, teriparatide has a detrimental effect on cortical bone in the short term [93], especially when switching from an antiresorptive treatment [94,95], and has been shown to amplify the negative effect of Dkk-1 [19,21]. Thus, the null effect of teriparatide on bone erosion was not surprising [96]. Nevertheless, teriparatide was shown to be extraordinarily effective in the treatment of glucocorticoid-induced OP [97] and, according to many international guidelines, should be the treatment of choice for patients at higher risk of fractures [59,60,61].

## 3. Spondyloarthritis and Psoriatic Arthritis

Pathological bone formation is one of the hallmarks of spondyloarthritis (SpA), including psoriatic arthritis (PsA) [98]. In long-term axial-SpA (ax-SpA), progressive ankylosis can develop and strongly contribute to the burden of the disease and resulting disability [99]. At the same time, OP and low BMD were shown to coexist and to represent a major comorbidity [100]. It is now known that inflammation at the spine can lead to local bone loss with increased risk of fracture, especially at the sites affected by bone marrow edema (BME)/osteitis [101,102]. In SpA, in contrast to inflammatory diseases (i.e., RA), GCs are less frequently used. The patient phenotype affected by bone loss seems to differ as well, since it has been reported to occur even in unexpected populations, such as in young males [103,104].

In the past, OP in SpA has been related with immobilization, due to the association between its severity, disease duration, and the radiographic scores of ankylosis [105]. However, this explanation seems to be incomplete, and bone loss was reported to be an early event in the course of the disease [106,107]. Indeed, spinal inflammation leads to trabecular bone loss and increased fracture risk. The presence of MRI lesions defined as BME/osteitis was reported to increase fivefold the risk of having a low spine BMD, and was also shown to be the single best determinant of low hip BMD [102]. The close relationship between BME and low BMD has been confirmed also in ax-SpA without radiographical involvement [108], once again supporting the earliness of this process. Another factor that was directly associated with osteoporosis and, at the same time, with spinal inflammation, is Dkk-1, a Wnt inhibitor [109]. To date, the available studies on the prevalence of vertebral fractures in patients with SpA show are quite heterogeneous [103], due to large differences in the examined populations and difficulties in their diagnosis [110,111,112]. The prevalence of VF in patients with SpA was reported to be from 0–4% to as high as 30–40% [112,113], with a greater risk (from threefold to sevenfold) than in the general population [104,114]. To complicate the picture, the definition of vertebral fracture also greatly varies across the studies [100,112]. In addition, treatment with TNF inhibitors has been associated with improved BMD but not with reduced incidence of vertebral fractures [115]. A recent meta-analysis confirmed these data, showing a range of 11.7 to 34.4%, 11.6 to 19.1%, and 6.4 to 16.8% for total OP, lumbar spine, and femoral neck OP measured by Dual-energy X-ray Absorptiometry (DXA), respectively [116]. In the same analysis, the prevalence of fracture ranged from 11% to 24.6%, with a high grade of heterogeneity for all outcomes [116].

When dealing with PsA, a study published in 2017 documented an elevated risk for incident fracture: 1.16 (95% CI 1.06–1.27) [117]. In the same study, patients with mild psoriasis were reported to be at similar risk for vertebral fractures, or at an even higher risk when patients with severe psoriasis were considered (mild psoriasis 1.07, 95% Confidence Interval [CI] 1.05–1.10, and severe psoriasis Hazard Ratio [HR] 1.26, 95 CI 1.15–1.39) [117].

To date, few studies have investigated the pathophysiology of bone loss in SpA. Unsurprisingly, the parameters of systemic inflammation (i.e., increase in the C-reactive protein) have also been associated with low BMD [107]. The role of systemic inflammation as a determinant for generalized bone loss is also supported by the demonstration of low cortical BMD, and the area and thickness measured by HRpQCT [118]; once again, this process was reported as an early event.

Genetic factors may play a role as well. Transgenic (TG) HLA B27 rats were shown to have lower mineral/matrix ratios and higher relative proteoglycan and advanced glycation end product compared to wild type [119], features that are associated with impaired bone-material properties.

A study on a similar model of TG HLA B27 rats also showed a higher loss of bone density and strength with progressing age [120]. This was also associated with increased bone remodeling in favor of bone resorption, as shown by a larger number of osteoclastic precursors in the bone marrow and a stronger osteoclastogenic response to RANKL or TNFα [120]. In a cohort of patients affected by SpA, dysregulation in the RANKL-RANK pathway was documented, with increased serum concentrations of RANKL (and higher expression of intracellular RANKL in CD4+ and CD8+ T cells) [121]. Furthermore, autoantibodies to OPG were dosed in a cohort of patients with SpA, and were shown to be associated with low hip BMD and fractures [122].

Overall, all these data seem to support the hypothesis that inflammation in SpA might be detrimental on bone health in two different ways: first, by causing generalized bone loss due to systemic negative effects (similar to what happens in other conditions such as psoriasis or inflammatory bowel diseases [123]), and second by locally affecting the sites involved in BME/osteitis.

To date, no double-blind, randomized clinical trial with antiresorptive or osteoanabolic agents has been conducted in the SpA population; therefore, the same recommendations for the treatment and management of postmenopausal and male OP are applied to the SpA population.

The effects of neridronate (an intravenous N-bisphosphonate with high skeletal affinity) have been investigated in terms of disease activity in an open-label study vs infliximab (a TNF inhibitor approved for the treatment of SpA) in patients affected by ankylosing spondylitis (AS) [124]. Neridronate was successful at reducing disease activity with additional benefits on BMD changes in the lumbar spine [124]. Another study on pamidronate (another intravenous bisphosphonate) vs golimumab (a TNF inhibitor approved for the treatment of SpA) showed similar improvements in the Bath Ankylosing Spondylitis Disease Activity Index (BASDAI) in the two groups, but with improvement in the Bath Ankylosing Spondylitis Functional Index (BASFI) and on MRI data on axial inflammation (evaluated according to Spondyloarthritis Research Consortium of Canada scoring system) being limited to the golimumab group [125].

Given the key role of inflammation and bone loss, anti-inflammatory drugs are expected to have a beneficial effect on bone. However, data on non-steroidal anti-inflammatory drugs (NSAIDs) are limited.

In a primary-care-based, nested case control study, the risk of any clinical fracture was decreased in patients with SpA taking NSAIDs [114]. In addition, data from a large population-based public health database support the protective role of NSAIDs on the risk of clinical fractures in patients with SpA, with an observed increased risk of fractures apparent only in those not on chronic NSAIDs [126].

When dealing with anti-TNF drugs, several meta-analyses have reported benefits in terms of BMD in patients with AS and PsA [127,128,129], but data on modifications of fracture incidence are still lacking.

In conclusion, bone loss is a relevant comorbidity factor in SpA patients, and must not be overlooked. Future studies on the possible benefits of osteoactive and anti-inflammatory agents (synthetic and biologic) in SpA patients need to focus on clinical and fracture outcomes in order to improve the treatment and management of this condition.

## 4. Systemic Lupus Erythematosus

Systemic Lupus Erythematosus (SLE) is a chronic autoimmune connective tissue disease that has a complex pathophysiology and a wide spectrum of clinical manifestations affecting potentially every tissue and system of the body [130]. Bone tissue is among them, and many factors linked to the disease and its treatment could exert a detrimental effect on it [131]. Moreover, bone is affected both at joint site, as erosions, and at a systemic level, with OP and fragility fractures [131,132]; in this section, we will focus on the latter.

OP and fracture risk in SLE are often underestimated and undertreated, especially if compared to RA, where they are better recognized as comorbidities. The last EULAR recommendations for SLE management took into consideration comorbidities such cardiovascular and infective risk, but they still did not mention OP evaluation and management [133,134].

An increased risk of fracture was reported by large population studies. Tedeschi et al. recently reported a three-fold increased risk of fracture among a population of 47,709 SLE patients, which was only partially attenuated by the correction for glucocorticoid use and dosage [135]. These results are similar to what was reported by Wang et al. on 14,544 SLE patients in whom fracture risk was twice that of matched controls. Moreover, those studies identified a higher fracture risk in young SLE patients compared to a healthy matched control. A British study on 7732 SLE patients reported an incidence rate ratio for OP 2.5 times higher than in controls [136].

The pathophysiology of bone loss in SLE is complex and multifactorial, accounting for traditional disease-related factors and treatment-related factors [131]. Osteoimmunology studies in inflammatory rheumatic disease demonstrated a strong influence of the immune system on bone cells like osteoblast, osteoclasts, and osteocytes. The effect of a disrupted inflammatory status, mainly driven by IL-1, IL-6, IL-17, and TNFalpha, is generally to stimulate osteoclast differentiation and activity on one hand, and to inhibit osteoblast activity on the other [16,137]. Those effects involve important pathways such as the Wnt system and the RANK-RANKL-OPG axis [18,138]. Polymorphism in RANKL and OPG genes was associated with increased vertebral fracture risk in premenopausal SLE patients [139].

SLE arthritis was shown in recent years to be much more erosive than described in the past, thanks to more detailed radiological techniques. Besides a low percentage of ACPA-positive patients with an RA overlap form, a significant number of SLE patients with erosive arthritis were reported to be positive for anticarbamilated protein antibodies (antiCarP) [140]. These antibodies were also found in RA and correlated to systemic bone loss as ACPA [141].

Another important factor is the metabolic one, especially the vitamin D and PTH axis. SLE patients are at increased risk of impairment of this system. All SLE patient are instructed to avoid sunlight and to use broad spectrum sunscreens [133,142]; these restrictions limits endogenous vitamin D production, reducing the circulating level of 25OH vitamin D. Moreover, patients with nephritis may have also a deficit of 1-25 hydroxylation of vitamin D, developing secondary hyperparathyroidism with decreased intestinal calcium absorption. A small study on juvenile SLE detected a low BMD in 15% of the population at the onset of the disease, the the authors found an association between low BMD and low serum levels of calcium and high serum levels of PTH [143]. Another study on adolescents with SLE reported that decreased BMD was associated with the absence of vitamin D supplementation [144]. A study published in 2007 on SLE adults also underlined an association between impaired renal function and low bone mass, but without evaluation of calcium metabolism [145]. Several studies, both cross-sectional and longitudinal, have indicated that vitamin D deficiency is prevalent among SLE patients, and that it is a risk factor for bone mass loss [146,147].

Sex hormones are a cornerstone for bone health, and their alteration is the basis of postmenopausal OP and age-related bone loss. Serum levels of dehydroandrostenedione (DHEA), a marker of adrenal androgen status, progressively decline from the third decade, accounting for age-related bone loss in the general population [148]. DHEA was described to be lower in SLE patients compared to control and in active SLE compared to quiescent [149]; moreover, lower levels have been associated with lower BMD in premenopausal SLE patients [150]. Medication can also influence hormonal levels. In particular, a high dose regimen of cyclophosphamide (an alkylating agent used for the treatment of cancer and autoimmune diseases) is associated with ovarian insufficiency, causing a premature beginning of menopause [151].

The prevalence of osteopenia was reported in a range from 25 to 74% of SLE patients, whereas OP from 1.4 to 68% [131]. The prevalence is variable, and depends on the type of population according to age, treatment, organ involvement, ongoing OP treatments, and notably, the definition of bone involvement. For example, in younger patients with secondary OP, the Z-score is much more accurate than T-score, although not all studies have made this distinction [152]. This variety is even more true for the prevalence of fractures, especially for vertebral ones, since vertebral fractures remain undetected due to a paucity of symptoms if not investigated properly with a screening technique such as vertebral morphometry. For the aforementioned reasons, it is important to consider the common young age of onset and the chronicity of the disease. In fact, low BMD and fragility vertebral fractures are present also in the pediatric population and premenopausal females, already in the early stages of the disease [143,144].

Bone turnover markers are used as surrogate outcomes of bone health in different contexts. For example, another study demonstrated that untreated, premenopausal SLE patients exhibited decreased levels of osteocalcin (a bone formation marker) and increased levels of C-terminal telopeptide of type-1 collagen (CTX, a bone resorption marker). Moreover, two other studies reported a similar observation on osteocalcin with, in addition, a correlation with disease activity; however, only one of them found an association between high levels of CTX and disease activity [153]. Lower P1NP levels were associated with a decline in BMD in premenopausal SLE patients. These changes in bone turnover markers could reflect the aforementioned effects of inflammation and other factors on bone turnover.

Other interesting data came from HRpQCT studies that have the advantage of being able to obtain precise information on cortical and trabecular bone selectively, not only data on density, but also on the microarchitecture of bone tissue. A few studies have investigated this novel technique, with most concluding that the impairment of cortical bone and, to a lesser extent, trabecular bone, is, in large part, linked to glucocorticoid use and disease activity. Juvenile SLE (jSLE) patients displayed prominent impairment of trabecular bone with low bone mass, without catch-up growth over time [154]. This impairment caused a reduction in bone mass peak, with a higher risk of OP in early adulthood [154]. Another study on jSLE described a reduction of the pQCT parameters of both trabecular and cortical bone, with a particular trabecular defect in those patients with vertebral fractures (12 out of 66 pediatric patients) [155].

Several studies and a few meta-analyses have studied the risk factors for OP and fragility fractures in SLE patients with conflicting results, at least partially, due to the presence of confounding variables (e.g., disease activity and type/intensity of treatment heterogeneity of disease manifestations in SLE, or of treatment protocol and outcome assessments). Studies are inconsistent regarding the correlation between disease activity and bone loss in SLE [145,156,157]. Studies failed to find an association that would, conversely, be better underlined by an association with SDI (index of damage) [155,157,158]. A Chinese study was able to find a correlation between BMD and the number of flares over a five-year period; this could be more reliable, considering the fluctuating nature of SLE activity [159]. On the other hand, studies reporting an association with damage score were influenced by the presence of fractures in the scoring system [155]. Moreover, some controversy exists around GCs [49,50]; even though a number of studies have reported them as risk factor for bone loss and fracture, a recent metaregression analysis failed to confirm an association between GC use and low BMD [160], in contrast with what was reported by Jacobs et al. in patients, assuming less than 7.5 mg/day [147].

A hallmark of SLE treatment is hydroxychloroquine (HCQ), which has a variety of properties; nevertheless, data on bone health are controversial. Two cross-sectional studies on female SLE patients reported higher lumbar and hip BMD among HCQ users compared to non-users [157,161]. In contrast, a six-year Dutch study showed a slight decrease in BMD with a positive association between hip BMD loss and HCQ exposure [147]. A similar Chinese study did not report any significant effect of HCQ on bone density [159]. It must be said that all these studies had quite small populations, with small BMD changes being observed over the time; additionally, not all possible confounders were evaluated, leading to weak conclusions on HCQ effect. A more recent paper by Cramarossa et al. on a much larger population but with a cross sectional design did not find any association between BMD and antimalarials [158].

The role of immunosuppressants is controversial, given that their use usually indicates the presence of a more severe form of SLE, making it difficult to clearly determine their role on bone. For example, one study associated cyclophosphamide use with greater risk of femoral neck fractures [162]. SLE patients on mofetil micofenolate (MMF, an immunosuppressant agent that inhibits inosine-5′-monophosphate dehydrogenase [IMPDH]) displayed normal osteoclastogenesis compared to SLE patient not taking MMF, showing a lower number of osteoclasts even if both populations exhibited similar serum RANKL levels [163]. The clinical meaning of this observation is not yet clear. Moreover, the cross-sectional study by Cramarossa on a large cohort of SLE patients failed to find a correlation between immunosuppressor use and BMD [158].

These data proved that OP and fragility fractures are a not rare complications of SLE, but rather, that they contribute substantially to disability and morbidity burden. Of interest, the management of hypertension and OP was proven to ameliorate organ damage accrual in SLE patients.

In conclusion, SLE patients must be screened for OP and for the presence of prevalent vertebral fracture. Risk factors must be corrected when possible, glucocorticoid use must be minimized, and vitamin D and calcium must be prescribed together with antiresorptives drugs when indicated, even in premenopausal women. It also must be considered that most of the present risk tools underestimate the risk in SLE patients, especially in premenopausal females and jSLE [49,50].

## 5. Systemic Sclerosis

Systemic sclerosis (SSc) is a connective tissue disease characterized by tissue fibrosis and microvascular involvement. Since it affects many organs and systems, OP has been hypothesized to be more frequent in this setting compared to the general population. Unfortunately, different methods have been used to report low bone mineral density, namely BMD expressed as g/cm^2^ or T- or Z-scores, so a definite prevalence of OP could not be given. In addition, SSc is a proteiform disease; thus, different study cohorts might have different characteristics. Nevertheless, some studies [164,165,166,167,168,169,170,171,172,173,174] have tried to evaluate OP prevalence, leading to the conclusion that SSc patients are at increased risk of OP. For example, one of the largest cohorts in the literature observed a prevalence of OP of 23.6% (CI 95%: 16.1–33.0%) [174]. In addition, the mean Z-score was significantly lower than 0, indicating a reduced bone density as compared to sex- and age-matched healthy subjects [174].

One of the possible reasons for the increased prevalence is that there are some OP risk factors that could be more frequent in SSc patients compared to the general population, namely, malabsorption secondary to gastro-intestinal involvement eventually leading to low weight, premature ovarian failure related to cyclophosphamide administration [167], hypothyroidism, which is a common feature associated with SSc [175,176], and systemic GCs; however, these medications are less frequently used than in other rheumatic disease such as arthritides. In addition, it was reported that SSc patients had significantly lower vitamin D levels compared to the general population [177,178,179]. Furthermore, it was hypothesized that supplementation may be less effective in this setting [180,181]. The data on specific disease characteristics being risk factors for OP are somewhat contrasting, with some studies finding a correlation with a longer disease duration or a diffuse pattern and others not [169,171,174]. There are many reasons for these controversies, such as different frequencies of treatment with steroids or vitamin D supplementation, different sample sizes, different methods to asses bone loss (BMD or T-score instead of the more reliable Z-scores in younger patients), or non-thorough evaluations of possible confounders (such as BMI).

When studying bone turnover markers in SSc, one should keep in mind that some of them are also involved in its pathogenesis. For example, the Wnt system has a well-known role in both fibrosis [182] and bone metabolism [183]; therefore, studying it in SSc is not easy, and possibly leads to conflicting results, as for Dkk-1. In fact, Ruaro et al. [184] found a correlation between elevated Dkk-1 and low Trabecular Bone Score (TBS), while Taylan [185] found a similar correlation with mRSS but not with BMD. Regarding the Wnt system, it is worth noting that it is strongly involved with low BMI-related OP [186], and in a previous study [174], a strong correlation was found between low BMI and OP. In contrast, data on the RANK-RANKL system are more concordant. In SSc patients higher serum levels of RANKL were found compared to a control group, while OPG levels were similar [185,187]. Interestingly, TRIAL, a ligand of OPG, also with vascular protection properties [188], has been found to be higher in SSc compared to the general population [185], suggesting a possible link between microvascular damage and bone loss.

Fewer data are available for mineral quality. Koumakis et al. [189] and Ruaro et al. [184] evaluated TBS in SSc, showing that patients had lower values compared to controls but similar to RA patients. Interestingly, Ruaro et al. showed that TBS was lower in patients with active or late videocapillaroscopy patterns. More expected was the correlation between GC treatment and low TBS, as shown by Koumakis, and between low TBS and a positive history of vertebral fractures, presented by Ruaro and colleagues.

Finally, data on fractures are limited to vertebral fractures and are quite variable, with a prevalence ranging between less than 15% [174,190] and up to 24–25% [191,192]. Interestingly, the majority of fractures involve the thoracic spine, but no effect on lung function was found [174].

## 6. Vasculitides

Osteoporosis in systemic vasculitides may occur secondary to the underlying vasculitis or treatment of the disease. Few data on osteoporosis and fracture risk are available for large and small vessel vasculitides.

Giant cell arteritis (GCA) is the most common systemic vasculitis among the elderly. Osteoporosis was present in 16% of GCA patients at study entry in a prospective, multicenter, longitudinal study that assessed damage accrual during time [193]. Moreover, GCs are still the mainstay of treatment of GCA. Hence, GCs-induced OP is a common comorbidity in patients with GCA. Clinical guidelines for GCA management suggest bone protection (bisphosphonate with calcium and vitamin D supplementation) for all patients [194]. A retrospective cohort study based in the UK calculated an incidence rate of fracture of 147.15 (132.91–162.91) in GCA per 10,000 person–years. Risk of fracture was increased by 67% (1.67, 1.49–1.88) compared to the control populations. However, among GCA patients with at least two prescriptions of GCs, only 10.1% were prescribed bisphosphonates [195].

Antineutrophil cytoplasmic antibody-associated vasculitis is a relapsing-remitting disease that has been shown to be associated with osteoporosis. A population-based cohort of patients with ANCA-associated vasculitides diagnosed between 1998 and 2010 in Southern Sweden was used to calculate the rate ratios for comorbidities compared with a reference population. The highest ratios were obtained for osteoporosis (4.6, 95% CI 3.0–7.0) [196]. Boomsa et al. demonstrated that among 99 patients with AAV, 57% percent had osteopenia, and 21% had osteoporosis at least at one site. Cumulative doses of GCs were significantly associated with bone loss at the spine and femur [197].

In addition, osteoporosis can be a long-term consequence of treatment in patients with AAV. In a prospective randomized trial by the French Vasculitis Study Group, 118 patients with polyarteritis nodosa or microscopic polyangiitis without poor-prognosis factors were assessed. At the last follow-up visit (mean 98.2 ± 41.9 months), the most frequent sequelae were peripheral neuropathy, hypertension, and osteoporosis in 16% of patients [198]. Long-term (up to 7 years) follow-up data available for patients from four combined European vasculitis study group trials demonstrated that patients with AAV develop osteoporosis over time, and osteoporosis was reported in 14.1% of the enrolled subjects (95% CI 9.9 to 18.2%) [199].

## 7. Sjogren Syndrome

Studies that evaluated BMD in primary Sjogren Syndrome (pSS) are scarce. One small study of 128 Chinese patients with pSS (86 post-menopausal) found osteopenia in 32.8% and osteoporosis in 51.6%. Only 6 patients had a history of fragility fracture [200]. Another retrospective study conducted in Taiwan recruited RA, SLE, and pSS patients undergoing bone mineral density DXA scans. Among the 118 patients with pSS, the cumulative incidence of osteoporotic fractures was 29.2%. The mean estimated 10-year risk of fragility fractures using FRAX tool varied between 4.6% for femoral fracture and 19.3% for major. The authors also argued that FRAX, either with or without BMD measurement, underestimates the fracture risk in pSS patients, even after stratification by age and glucocorticoid treatment [201].

## 8. Crystal-Induced Arthritides

Crystal-induced arthritides are inflammatory joint diseases caused by the deposition of crystals within the joints. They are characterized by acute episodes of inflammation in joints, mainly driven by proinflammatory cytokines such as interleukin (IL)-1 and IL-6. Prior studies suggest an association between osteoporosis, systemic inflammation, and proinflammatory cytokines.

Calcium pyrophosphate deposition disease (CPDD) is a common cause of acute and chronic crystal-induced arthritis, but evidence in the literature of the association with osteoporosis is scarce. In the largest epidemiologic study of 25,157 patients with CPDD from the Department of VA Corporate Data Warehouse, a positive, albeit modest, association with osteoporosis was found (OR 1.26 [95% CI 1.16–1.36]) [202].

Likewise, it remains unclear whether gout is associated with osteoporosis, or it affects fracture risk. Gout is the among the most common crystal-induced arthritides, and is characterized by hyperuricemia, leading to crystallization of serum uric acid (sUA) in joints and tendons, resulting in systemic inflammation with oxidative stress and bone micro-inflammation. Nevertheless, sUA might have a protective effect on BMD [203,204,205,206]. Indeed, Mendelian randomization analyses did not support a causal effect of sUA on low BMD [207,208].

A nationwide, population-based, retrospective, matched-cohort study in Taiwan compared two matched cohorts (n = 36,458 with gout and 71,602 without) recruited from the Longitudinal Health Insurance Dataset. Incidence ratio for osteoporosis were calculated, excluding participants with osteoporosis before study entry and controlling for several confounders, in particular glucocorticoid use, allopurinol exposure, and other comorbidities. This study further supports the hypothesis that patients with gout have a modestly-increased risk of developing osteoporosis compared to individuals without the disease [209].

However, there is not convincing evidence that gout is associated with increased risk of fractures. Data from a US commercial health plan (2004–2013) on 73,202 gout and 219,606 non-gout patients, matched by age, sex, and date of study entry calculated an adjusted hazard ratio (HR) of 0.98 (95% confidence interval [CI] 0.85–1.12) for non-vertebral fracture and 0.83 (95% CI 0.65–1.07) for hip fracture in gout versus non-gout. Moreover, subgroup analyses showed no association between baseline sUA and non-vertebral fracture (HR = 1.03, 95% CI 0.93–1.15), adjusted for age, sex, comorbidity score, and number of prescription drugs, which was confirmed also among patients with gout [210].

In summary, since quality evidence is lacking, further studies investigating the association between gout, hyperuricemia, and osteoporosis are urgently needed.

## 9. Conclusions

Rheumatic diseases represent a wide spectrum of conditions characterized by the inflammation and destruction of various structures of the body. Bone tissue is commonly involved in many rheumatic diseases, and osteoporosis represents the most frequent bone disease in rheumatic conditions. Rheumatologists and general practitioners should be aware of the increased risk of OP in rheumatic patients, especially because patients affected by rheumatic diseases have access to several anti-osteoporotic medications with proven efficacy and safety. However, the selection of the correct anti-osteoporotic treatment should be made on a case-by-case basis, taking into consideration the severity of OP and the underlying condition that is causing it.

## References

[1] Smith E., Hoy D.G., Cross M., Vos T., Naghavi M., Buchbinder R., Woolf A.D., March L. (2014). The global burden of other musculoskeletal disorders: Estimates from the Global Burden of Disease 2010 study. Ann. Rheum. Dis..

[2] Rossini M., Gatti D., Adami S. (2013). Involvement of WNT/β-catenin signaling in the treatment of osteoporosis. Calcif. Tissue Int..

[3] Lacey D.L., Boyle W.J., Simonet W.S., Kostenuik P.J., Dougall W.C., Sullivan J.K., San Martin J., Dansey R. (2012). Bench to bedside: Elucidation of the OPG-RANK-RANKL pathway and the development of denosumab. Nat. Rev. Drug Discov..

[4] Saag K.G., Petersen J., Brandi M.L., Karaplis A.C., Lorentzon M., Thomas T., Maddox J., Fan M., Meisner P.D., Grauer A. (2017). Romosozumab or Alendronate for Fracture Prevention in Women with Osteoporosis. N. Engl. J. Med..

[5] Rodan G.A., Fleisch H.A. (1996). Bisphosphonates: Mechanisms of action. J. Clin. Investig..

[6] Cummings S.R., Ferrari S., Eastell R., Gilchrist N., Jensen J.-E.B., McClung M., Roux C., Törring O., Valter I., Wang A.T. (2018). Vertebral Fractures After Discontinuation of Denosumab: A Post Hoc Analysis of the Randomized Placebo-Controlled FREEDOM Trial and Its Extension. J. Bone Miner. Res..

[7] Makino A., Takagi H., Takahashi Y., Hase N., Sugiyama H., Yamana K., Kobayashi T. (2018). Abaloparatide Exerts Bone Anabolic Effects with Less Stimulation of Bone Resorption-Related Factors: A Comparison with Teriparatide. Calcif. Tissue Int..

[8] McInnes I.B., Schett G. (2011). The Pathogenesis of Rheumatoid Arthritis. New Engl. J. Med..

[9] Adami G., Saag K.G. (2019). Osteoporosis Pathophysiology, Epidemiology, and Screening in Rheumatoid Arthritis. Curr. Rheumatol. Rep..

[10] Kleyer A., Finzel S., Rech J., Manger B., Krieter M., Faustini F., Araujo E., Hueber A.J., Harre U., Engelke K. (2014). Bone Loss Before the Clinical Onset of Rheumatoid Arthritis in Subjects with Anticitrullinated Protein Antibodies. Ann. Rheum. Dis..

[11] Haugeberg G., Helgetveit K.B., Forre O., Garen T., Sommerseth H., Proven A. (2014). Generalized Bone Loss in Early Rheumatoid Arthritis Patients Followed for Ten Years in the Biologic Treatment Era. BMC Musculoskelet. Disord..

[12] Zhu T.Y., Griffith J.F., Qin L., Hung V.W., Fong T.N., Au S.K., Li M., Lam Y.Y.O., Wong C.K., Kwok A.W. (2014). Alterations of Bone Density, Microstructure, and Strength of the Distal Radius in Male Patients with Rheumatoid Arthritis: A Case-Control Study with HR-pQCT. J. Bone Miner. Res..

[13] Simon D., Kleyer A., Stemmler F., Simon C., Berlin A., Hueber A.J., Haschka J., Renner N., Figueiredo C., Neuhuber W. (2017). Age- And Sex-Dependent Changes of Intra-Articular Cortical and Trabecular Bone Structure and the Effects of Rheumatoid Arthritis. J. Bone Miner. Res..

[14] Stemmler F., Simon D., Liphardt A.M., Englbrecht M., Rech J., Hueber A.J., Engelke K., Schett G., Kleyer A. (2018). Biomechanical Properties of Bone are Impaired in Patients with ACPA-Positive Rheumatoid Arthritis and Associated with the Occurrence of Fractures. Ann. Rheum. Dis..

[15] Black D.M., Rosen C.J. (2016). Clinical Practice. Postmenopausal Osteoporosis. N. Engl. J. Med..

[16] Takayanagi H. (2012). New Developments in Osteoimmunology. Nat. Rev. Rheumatol..

[17] Fessler J., Husic R., Schwetz V., Lerchbaum E., Aberer F., Fasching P., Ficjan A., Obermayer-Pietsch B., Duftner C., Graninger W. (2018). Senescent T-Cells Promote Bone Loss in Rheumatoid Arthritis. Front. Immunol..

[18] Diarra D., Stolina M., Polzer K., Zwerina J., Ominsky M.S., Dwyer D., Korb A., Smolen J., Hoffmann M., Scheinecker C. (2007). Dickkopf-1 is a Master Regulator of Joint Remodeling. Nat. Med..

[19] Rossini M., Viapiana O., Adami S., Fracassi E., Idolazzi L., Dartizio C., Povino M.R., Orsolini G., Gatti D. (2015). In Patients with Rheumatoid Arthritis, Dickkopf-1 Serum Levels are Correlated with Parathyroid Hormone, Bone Erosions and Bone Mineral Density. Clin. Exp. Rheumatol..

[20] Ma Y., Zhang X., Wang M., Xia Q., Yang J., Wu M., Han R., Chen M., Hu X., Yuan Y. (2018). The Serum Level of Dickkopf-1 in Patients with Rheumatoid Arthritis: A Systematic Review and Meta-Analysis. Int. Immunopharmacol..

[21] Gatti D., Viapiana O., Idolazzi L., Fracassi E., Rossini M., Adami S. (2011). The Waning of Teriparatide Effect on Bone Formation Markers in Postmenopausal Osteoporosis is Associated with Increasing Serum Levels of DKK1. J. Clin. Endocrinol. Metab..

[22] Viapiana O., Fracassi E., Troplini S., Idolazzi L., Rossini M., Adami S., Gatti D. (2013). Sclerostin and DKK1 in Primary Hyperparathyroidism. Calcif. Tissue Int..

[23] Orsolini G., Adami G., Rossini M., Ghellere F., Caimmi C., Fassio A., Idolazzi L., Gatti D., Viapiana O. (2018). Parathyroid Hormone is a Determinant of Serum Dickkopf-1 Levels in Ankylosing Spondylitis. Clin. Rheumatol..

[24] Adami G., Rossini M., Bogliolo L., Cantatore F.P., Varenna M., Malavolta N., Puente A.D., Muratore M., Orsolini G., Gatti D. (2018). An Exploratory Study on the Role of Vitamin D Supplementation in Improving Pain and Disease Activity in Rheumatoid Arthritis. Mod. Rheumatol..

[25] Brance M.L., Brun L.R., Lioi S., Sanchez A., Abdala M., Oliveri B. (2015). Vitamin D Levels and Bone Mass in Rheumatoid Arthritis. Rheumatol. Int..

[26] Rossini M., Adami G., Viapiana O., Idolazzi L., Orsolini G., Fassio A., Giollo A., Gatti D. (2017). Osteoporosis: An Independent Determinant of Bone Erosions in Rheumatoid Arthritis?. J. Bone Miner. Res..

[27] Kocijan R., Harre U., Schett G. (2013). ACPA and Bone Loss in Rheumatoid Arthritis. Curr. Rheumatol. Rep..

[28] Orsolini G., Viapiana O., Rossini M., Adami G., Caimmi C., Fassio A., Gatti D. (2018). Anti-CCP Antibodies and Bone. Arthritis Res. Ther..

[29] Orsolini G., Caimmi C., Viapiana O., Idolazzi L., Fracassi E., Gatti D., Adami G., Rossini M. (2017). Titer-Dependent Effect of Anti-Citrullinated Protein Antibodies on Systemic Bone Mass in Rheumatoid Arthritis Patients. Calcif. Tissue Int..

[30] Bugatti S., Bogliolo L., Vitolo B., Manzo A., Montecucco C., Caporali R. (2016). Anti-Citrullinated Protein Antibodies and High Levels of Rheumatoid Factor are Associated with Systemic Bone Loss in Patients with Early Untreated Rheumatoid Arthritis. Arthritis Res. Ther..

[31] Simon D., Kleyer A., Englbrecht M., Stemmler F., Simon C., Berlin A., Kocijan R., Haschka J., Hirschmann S., Atreya R. (2018). A Comparative Analysis of Articular Bone in Large Cohort of Patients with Chronic Inflammatory Diseases of the Joints, the Gut and the skin. Bone.

[32] Behrens F., Koehm M., Thaçi D., Gnann H., Greger G., Maria Wittig B., Burkhardt H. (2016). Anti-Citrullinated Protein Antibodies are Linked to Erosive Disease in an Observational Study of Patients with Psoriatic Arthritis. Rheumatology.

[33] Haugeberg G., Uhlig T., Falch J.A., Halse J.I., Kvien T.K. (2000). Bone Mineral Density and Frequency of Osteoporosis in Female Patients with Rheumatoid Arthritis: Results from 394 Patients in the Oslo County Rheumatoid Arthritis Register. Arthritis Rheum..

[34] Sinigaglia L., Nervetti A., Mela Q., Bianchi G., Del Puente A., Di Munno O., Frediani B., Cantatore F., Pellerito R., Bartolone S. (2000). A multicenter Cross Sectional Study on Bone Mineral Density in Rheumatoid Arthritis. Italian Study Group on Bone Mass in Rheumatoid Arthritis. J. Rheumatol..

[35] Hauser B., Riches P.L., Wilson J.F., Horne A.E., Ralston S.H. (2014). Prevalence and Clinical Prediction of Osteoporosis in a Contemporary Cohort of Patients with Rheumatoid Arthritis. Rheumatology.

[36] Mori Y., Kuwahara Y., Chiba S., Kogre A., Baba K., Kamimura M., Itoi E. (2017). Bone Mineral Density of Postmenopausal Women with Rheumatoid Arthritis Depends on Disease Duration Regardless of Treatment. J. Bone Miner. Metab..

[37] Fassio A., Idolazzi L., Jaber M.A., Dartizio C., Viapiana O., Rossini M., Gatti D. (2016). The negative bone Effects of the Disease and of Chronic Corticosteroid Treatment in Premenopausal Women Affected by Rheumatoid Arthritis. Reumatismo.

[38] Kweon S.M., Sohn D.H., Park J.H., Koh J.H., Park E.K., Lee H.N., Kim K., Kim Y., Kim G.T., Lee S.G. (2018). Male Patients with Rheumatoid Arthritis Have an Increased Risk of Osteoporosis: Frequency and Risk Factors. Medicine.

[39] Jin S., Hsieh E., Peng L., Yu C., Wang Y., Wu C., Wang Q., Li M., Zeng X. (2018). Incidence of Fractures Among Patients with Rheumatoid Arthritis: A Systematic Review and Meta-Analysis. Osteoporos. Int..

[40] Xue A.L., Wu S.Y., Jiang L., Feng A.M., Guo H.F., Zhao P. (2017). Bone Fracture Risk in Patients with Rheumatoid Arthritis: A meta-Analysis. Medicine.

[41] Chen B., Cheng G., Wang H., Feng Y. (2016). Increased Risk of Vertebral Fracture in Patients with Rheumatoid Arthritis: A Meta-Analysis. Medicine.

[42] Dougados M., Soubrier M., Antunez A., Balint P., Balsa A., Buch M.H., Casado G., Detert J., El-Zorkany B., Emery P. (2014). Prevalence of Comorbidities in Rheumatoid Arthritis and Evaluation of Their Monitoring: Results of an International, Cross-Sectional Study (COMORA). Ann. Rheum. Dis..

[43] Richards J.S., Cannon G.W., Hayden C.L., Amdur R.L., Lazaro D., Mikuls T.R., Reimold A.M., Caplan L., Johnson D.S., Schwab P. (2012). Adherence with Bisphosphonate Therapy in US Veterans with Rheumatoid Arthritis. Arthritis Care Res..

[44] Ozen G., Kamen D.L., Mikuls T.R., England B.R., Wolfe F., Michaud K. (2018). Trends and Determinants of Osteoporosis Treatment and Screening in patients with Rheumatoid Arthritis Compared to Osteoarthritis. Arthritis Care Res..

[45] Chatzidionysiou K., Emamikia S., Nam J., Ramiro S., Smolen J., Van Der Heijde D., Dougados M., Bijlsma J., Burmester G., Scholte M. (2017). Efficacy of Glucocorticoids, Conventional and Targeted Synthetic Disease-Modifying Antirheumatic Drugs: A Systematic Literature Review Informing the 2016 Update of the EULAR Recommendations for the Management of Rheumatoid Arthritis. Ann. Rheum. Dis..

[46] Saag K.G., Koehnke R., Caldwell J.R., Brasington R., Burmeister L.F., Zimmerman B., Kohler J.A., Furst D.E. (1994). Low Dose Long-Term Corticosteroid Therapy in Rheumatoid Arthritis: An Analysis of Serious Adverse Events. Am. J. Med..

[47] Balasubramanian A., Wade S.W., Adler R.A., Lin C.J.F., Maricic M., O’Malley C.D., Saag K., Curtis J.R. (2016). Glucocorticoid Exposure and Fracture Risk in Patients with New-Onset Rheumatoid Arthritis. Osteoporos. Int..

[48] Balasubramanian A., Wade S.W., Adler R.A., Saag K., Pannacciulli N., Curtis J.R. (2018). Glucocorticoid Exposure and Fracture Risk in a Cohort of US Patients with Selected Conditions. J. Bone Miner. Res..

[49] Adami G., Saag K.G. (2019). Glucocorticoid-Induced Osteoporosis Update. Curr. Opin. Rheumatol..

[50] Adami G., Saag K.G. (2019). Glucocorticoid-Induced Osteoporosis: 2019 Concise Clinical Review. Osteoporos. Int..

[51] Adami G., Rahn E.J., Saag K.G. (2019). Glucocorticoid-Induced Osteoporosis: From Clinical Trials to Clinical Practice. Ther. Adv. Musculoskelet..

[52] Sambrook P.N., Eisman J.A., Yeates M.G., Pocock N.A., Eberl S., Champion G.D. (1986). Osteoporosis in Rheumatoid Arthritis: Safety of Low Dose Corticosteroids. Ann. Rheum. Dis..

[53] Blavnsfeldt A.B.G., De Thurah A., Thomsen M.D., Tarp S., Langdahl B., Hauge E.M. (2018). The Effect of Glucocorticoids on Bone Mineral Density in Patients with Rheumatoid Arthritis: A Systematic Review and Meta-Analysis of Randomized, Controlled Trials. Bone.

[54] Siu S., Haraoui B., Bissonnette R., Bessette L., Roubille C., Richer V., Starnino T., McCourt C., McFarlane A., Fleming P. (2015). Meta-Analysis of Tumor Necrosis Factor Inhibitors and Glucocorticoids on Bone Density in Rheumatoid Arthritis and Ankylosing Spondylitis Trials. Arthritis Care Res..

[55] Baillet A., Gossec L., Carmona L., Wit M., De Van Eijk-Hustings Y., Bertheussen H., Alison K., Toft M., Kouloumas M., Ferreira R.J.O. (2016). Points to Consider for Reporting, Screening for and Preventing Selected Comorbidities in Chronic Inflammatory Rheumatic Diseases in Daily Practice: A Eular Initiative. Ann. Rheum. Dis..

[56] Kanis J.A., Johnell O., Oden A., Johansson H., McCloskey E. (2008). FRAX and the Assessment of Fracture Probability in Men and Women from the UK. Osteoporos. Int..

[57] Devogelaer J.P., Goemaere S., Boonen S., Body J.J., Kaufman J.M., Reginster J.Y., Rozenberg S., Boutsen Y. (2006). Evidence-Based Guidelines for the Prevention and Treatment of Glucocorticoid-Induced Osteoporosis: A Consensus Document of the Belgian Bone Club. Osteoporos. Int..

[58] Suzuki Y., Nawata H., Soen S., Fujiwara S., Nakayama H., Tanaka I., Ozono K., Sagawa A., Takayanagi R., Tanaka H. (2014). Guidelines on the Management and Treatment of Glucocorticoid-Induced Osteoporosis of the Japanese Society for Bone and Mineral Research: 2014 update. J. Bone Miner. Metab..

[59] Rossini M., Adami S., Bertoldo F., Diacinti D., Gatti D., Giannini S., Giusti A., Malavolta N., Minisola S., Osella G. (2016). Guidelines for the Diagnosis, Prevention and Management of Osteoporosis. Reumatismo.

[60] Buckley L., Guyatt G., Fink H.A., Cannon M., Grossman J., Hansen K.E., Humphrey M.B., Lane N.E., Magrey M., Miller M. (2017). 2017 American College of Rheumatology Guideline for the Prevention and Treatment of Glucocorticoid-Induced Osteoporosis. Arthritis Rheumatol..

[61] Compston J., Cooper A., Cooper C., Gittoes N., Gregson C., Harvey N., Hope S., Kanis J.A., McCloskey E.V., Poole K.E.S. (2017). UK Clinical Guideline for the Prevention and Treatment of Osteoporosis. Arch. Osteoporos..

[62] Fassio A., Rossini M., Viapiana O., Idolazzi L., Vantaggiato E., Benini C., Gatti D. (2017). New Strategies for the Prevention and Treatment of Systemic and Local Bone Loss; from Pathophysiology to Clinical Application. Curr. Pharm. Des..

[63] Barreira S.C., Fonseca J.E. (2016). The Impact of Conventional and Biological Disease Modifying Antirheumatic Drugs on Bone Biology. Rheumatoid Arthritis as a Case Study. Clin. Rev. Allergy Immunol..

[64] Zerbini C.A.F., Clark P., Mendez-Sanchez L., Pereira R.M.R., Messina O.D., Una C.R., Adachi J.D., Lems W.F., Cooper C., Lane N.E. (2017). Biologic Therapies and Bone Loss in Rheumatoid Arthritis. Osteoporos. Int..

[65] Manara M., Sinigaglia L. (2015). Bone and TNF in Rheumatoid Arthritis: Clinical Implications. RMD Open.

[66] Orsolini G., Fassio A., Rossini M., Adami G., Giollo A., Caimmi C., Idolazzi L., Viapiana O., Gatti D. (2019). Effects of Biological and Targeted Synthetic DMARDs on Bone Loss in Rheumatoid Arthritis. Pharmacol. Res..

[67] Orsolini G., Adami G., Adami S., Viapiana O., Idolazzi L., Gatti D., Rossini M. (2016). Short-Term Effects of TNF Inhibitors on Bone Turnover Markers and Bone Mineral Density in Rheumatoid Arthritis. Calcif. Tissue Int..

[68] Adami G., Orsolini G., Adami S., Viapiana O., Idolazzi L., Gatti D., Rossini M. (2016). Effects of TNF Inhibitors on Parathyroid Hormone and Wnt Signaling Antagonists in Rheumatoid Arthritis. Calcif. Tissue Int..

[69] Vis M., Havaardsholm E.A., Haugeberg G., Uhlig T., Voskuyl A.E., Van De Stadt R.J., Dijkmans B.A.C., Woolf A.D., Kvien T.K., Lems W.F. (2006). Evaluation of Bone Mineral Density, Bone Metabolism, Osteoprotegerin and Receptor Activator of the NFkappaB ligand Serum Levels During Treatment with Infliximab in Patients with Rheumatoid Arthritis. Ann. Rheum. Dis..

[70] Szentpetery A., McKenna M.J., Murray B.F., Ng C.T., Brady J.J., Morrin M., Radovits B., Veale D.J., Fitzgerald O. (2013). Periarticular Bone Gain at Proximal Interphalangeal Joints and Changes in Bone Turnover Markers in Response to Tumor Necrosis Factor Inhibitors in Rheumatoid and Psoriatic Arthritis. J. Rheumatol..

[71] Fassio A., Adami G., Gatti D., Orsolini G., Giollo A., Idolazzi L., Benini C., Vantaggiato E., Rossini M., Viapiana O. (2018). Inhibition of Tumor Necrosis Factor-Alpha (TNF-alpha) in Patients with Early Rheumatoid Arthritis Results in Acute Changes of Bone Modulators. Int. Immunopharmacol..

[72] Ozen G., Pedro S., Wolfe F., Michaud K. (2019). Medications Associated with Fracture Risk in Patients with Rheumatoid Arthritis. Ann. Rheum. Dis..

[73] Tada M., Inui K., Sugioka Y., Mamoto K., Okano T., Koike T. (2018). Abatacept Might Increase Bone Mineral Density at Femoral Neck for Patients with Rheumatoid Arthritis in Clinical Practice: Airtight Study. Rheumatol. Int..

[74] Chen Y.M., Chen H.H., Huang W.N., Liao T.L., Chen J.P., Chao W.C., Lin C.T., Hung W.T., Hsieh C.W., Hsieh T.Y. (2017). Tocilizumab Potentially Prevents Bone Loss in Patients with Anticitrullinated Protein Antibody-Positive Rheumatoid Arthritis. PLoS ONE.

[75] Wheater G., Elshahaly M., Naraghi K., Tuck S.P., Datta H.K., Van Laar J.M. (2018). Changes in Bone Density and Bone Turnover in Patients with Rheumatoid Arthritis Treated with Rituximab, Results from an Exploratory, Prospective Study. PLoS ONE.

[76] Sanderson J., Martyn-St James M., Stevens J., Goka E., Wong R., Campbell F., Selby P., Gittoes N., Davis S. (2016). Clinical Effectiveness of Bisphosphonates for the Prevention of Fragility Fractures: A Systematic Review and Network Meta-Analysis. Bone.

[77] Rossini M., Adami G., Adami S., Viapiana O., Gatti D. (2016). Safety Issues and Adverse Reactions with Osteoporosis Management. Expert Opin. Drug Saf..

[78] Saag K.G., Emkey R., Schnitzer T.J., Brown J.P., Hawkins F., Goemaere S., Thamsborg G., Liberman U.A., Delmas P.D., Malice M.P. (1998). Alendronate for the Prevention and Treatment of Glucocorticoid-Induced Osteoporosis. Glucocorticoid-Induced Osteoporosis Intervention Study Group. N. Engl. J. Med..

[79] Reid D.M., Devogelaer J.P., Saag K., Roux C., Lau C.S., Reginster J.Y., Papanastasiou P., Ferreira A., Hartl F., Fashola T. (2009). Zoledronic Acid and Risedronate in the Prevention and Treatment of Glucocorticoid-Induced Osteoporosis (HORIZON): A Multicentre, Double-Blind, Double-Dummy, Randomised Controlled Trial. Lancet.

[80] Cohen S., Levy R.M., Keller M., Boling E., Emkey R.D., Greenwald M., Zizic T.M., Wallach S., Sewell K.L., Lukert B.P. (1999). Risedronate Therapy Prevents Corticosteroid-Induced Bone Loss: A Twelve-Month, Multicenter, Randomized, Double-Blind, Placebo-Controlled, Parallel-Group Study. Arthritis Rheum..

[81] Ringe J.D., Dorst A., Faber H., Ibach K., Sorenson F. (2003). Intermittent Intravenous Ibandronate Injections Reduce Vertebral Fracture Risk in Corticosteroid-Induced Osteoporosis: Results from a Long-Term Comparative Study. Osteoporos. Int..

[82] Jarrett S.J., Conaghan P.G., Sloan V.S., Papanastasiou P., Ortmann C.E., O’Connor P.J., Grainger A.J., Emery P. (2006). Preliminary Evidence for a Structural Benefit of the New Bisphosphonate Zoledronic Acid in Early Rheumatoid Arthritis. Arthritis Rheum..

[83] Rossini M., Adami G., Viapiana O., Idolazzi L., Fassio A., Giollo A., Caimmi C., Orsolini G., Gatti D. (2018). Rheumatoid Arthritis, γδ T Cells and Bisphosphonates. Ann. Rheum. Dis..

[84] Cummings S.R., San Martin J., McClung M.R., Siris E.S., Eastell R., Reid I.R., Delmas P., Zoog H.B., Austin M., Wang A. (2009). Denosumab for Prevention of Fractures in Postmenopausal Women with Osteoporosis. N. Engl. J. Med..

[85] Saag K.G., Wagman R.B., Geusens P., Adachi J.D., Messina O.D., Emkey R., Chapurlat R., Wang A., Pannacciulli N., Lems W.F. (2018). Denosumab Versus Risedronate in Glucocorticoid-Induced Osteoporosis: A Multicentre, Randomised, Double-Blind, Active-Controlled, Double-Dummy, Non-Inferiority Study. Lancet Diabetes Endocrinol..

[86] Zebaze R., Libanati C., McClung M.R., Zanchetta J.R., Kendler D.L., Hoiseth A., Wang A., Ghasem-Zadeh A., Seeman E. (2016). Denosumab Reduces Cortical Porosity of the Proximal Femoral Shaft in Postmenopausal Women with Osteoporosis. J. Bone Miner. Res..

[87] Sharp J.T., Tsuji W., Ory P., Harper-Barek C., Wang H., Newmark R. (2010). Denosumab Prevents Metacarpal Shaft Cortical Bone Loss in Patients with Erosive Rheumatoid Arthritis. Arthritis Care Res..

[88] Rossini M., Adami G., Viapiana O., Idolazzi L., Gatti D. (2016). Denosumab, Cortical Bone and Bone Erosions in Rheumatoid Arthritis. Ann. Rheum. Dis..

[89] Cohen S.B., Dore R.K., Lane N.E., Ory P.A., Peterfy C.G., Sharp J.T., Van Der Heijde D., Zhou L., Tsuji W., Newmark R. (2008). Denosumab Treatment Effects on Structural Damage, Bone Mineral Density, and Bone Turnover in Rheumatoid Arthritis: A Twelve-Month, Multicenter, Randomized, Double-Blind, Placebo-Controlled, Phase Ii Clinical Trial. Arthritis Rheum..

[90] Takeuchi T., Tanaka Y., Ishiguro N., Yamanaka H., Yoneda T., Ohira T., Okubo N., Genant H.K., Van Der Heijde D. (2016). Effect of Denosumab on Japanese Patients with Rheumatoid Arthritis: A Dose-Response Study of AMG 162 (Denosumab) in Patients with RheumatoId Arthritis on Methotrexate to Validate Inhibitory Effect on Bone Erosion (DRIVE)-a 12-Month, Multicentre, Randomised, Double-Blind, Placebo-Controlled, Phase II Clinical Trial. Ann. Rheum. Dis..

[91] Ebina K., Hirao M., Hashimoto J., Matsuoka H., Iwahashi T., Chijimatsu R., Etani Y., Okamura G., Miyama A., Yoshikawa H. (2018). Impact of Switching Oral Bisphosphonates to Denosumab or Daily Teriparatide on the Progression of Radiographic Joint Destruction in Patients with Biologic-Naive Rheumatoid Arthritis. Osteoporos. Int..

[92] Solomon D.H., Kay J., Duryea J., Lu B., Bolster M.B., Yood R.A., Han R., Ball S., Coleman C., Lo E. (2017). Effects of Teriparatide on Joint Erosions in Rheumatoid Arthritis: A Randomized Controlled Trial. Arthritis Rheumatol..

[93] Ebina K., Hashimoto J., Shi K., Kashii M., Hirao M., Yoshikawa H. (2014). Comparison of the Effect of 18-Month Daily Teriparatide Administration on Patients with Rheumatoid Arthritis and Postmenopausal Osteoporosis Patients. Osteoporos. Int..

[94] Tsai J.N., Nishiyama K.K., Lin D., Yuan A., Lee H., Bouxsein M.L., Leder B.Z. (2017). Effects of Denosumab and Teriparatide Transitions on Bone Microarchitecture and Estimated Strength: The DATA-Switch HR-pQCT study. J. Bone Miner. Res..

[95] Idolazzi L., Rossini M., Viapiana O., Braga V., Fassio A., Benini C., Kunnathully V., Adami S., Gatti D. (2016). Teriparatide and Denosumab Combination Therapy and Skeletal Metabolism. Osteoporos. Int..

[96] Adami G., Rossini M., Viapiana O., Fassio A., Idolazzi L., Orsolini G., Gatti D. (2018). Lack of Effect of Teriparatide on Joint Erosions in Rheumatoid Arthritis Is an Expected Result: Comment on the Article by Solomon et al. Arthritis Rheumatol..

[97] Saag K.G., Shane E., Boonen S., Marin F., Donley D.W., Taylor K.A., Dalsky G.P., Marcus R. (2007). Teriparatide or Alendronate in Glucocorticoid-Induced Osteoporosis. New Engl. J. Med..

[98] Dougados M., Baeten D. (2011). Spondyloarthritis. Lancet.

[99] Machado P., Landewe R., Braun J., Hermann K.G.A., Baker D., Van Der Heijde D. (2010). Both Structural Damage and Inflammation of the Spine Contribute to Impairment of Spinal Mobility in Patients with Ankylosing Spondylitis. Ann. Rheum. Dis..

[100] Molto A., Etcheto A., Van Der Heijde D., Landewe R., Van Den Bosch F., Bautista Molano W., Burgos-Vargas R., Cheung P.P., Collantes-Estevez E., Deodhar A. (2016). Prevalence of Comorbidities and Evaluation of Their Screening in Spondyloarthritis: Results of the International Cross-Sectional ASAS-COMOSPA Study. Ann. Rheum. Dis..

[101] Maas F., Spoorenberg A., Van Der Slik B.P.G., Van Der Veer E., Brouwer E., Bootsma H., Bos R., Wink F.R., Arends S. (2017). Clinical Risk Factors for the Presence and Development of Vertebral Fractures in Patients with Ankylosing Spondylitis. Arthritis Care Res..

[102] Briot K., Durnez A., Paternotte S., Miceli-Richard C., Dougados M., Roux C. (2013). Bone Oedema on MRI is Highly Associated with Low Bone Mineral Density in Patients with Early Inflammatory Back Pain: Results from the DESIR Cohort. Ann. Rheum. Dis..

[103] Roux C. (2011). Osteoporosis in Inflammatory Joint Diseases. Osteoporos. Int..

[104] Weiss R.J., Wick M.C., Ackermann P.W., Montgomery S.M. (2010). Increased Fracture Risk in Patients with Rheumatic Disorders and Other Inflammatory Diseases—A Case-Control Study with 53,108 Patients with Fracture. J. Rheumatol..

[105] Ghozlani I., Ghazi M., Nouijai A., Mounach A., Rezqi A., Achemlal L., Bezza A., El Maghraoui A. (2009). Prevalence and Risk Factors of Osteoporosis and Vertebral Fractures in Patients with Ankylosing Spondylitis. Bone.

[106] Will R., Palmer R., Bhalla A.K., Ring F., Calin A. (1989). Osteoporosis in Early Ankylosing Spondylitis: A Primary Pathological Event?. Lancet.

[107] Van Der Weijden M.A.C., Claushuis T.A.M., Nazari T., Lems W.F., Dijkmans B.A.C., Van Der Horst-Bruinsma I.E. (2012). High Prevalence of Low Bone Mineral Density in Patients within 10 Years of Onset of Ankylosing Spondylitis: A Systematic Review. Clin. Rheumatol..

[108] Akgol G., Kamanlı A., Ozgocmen S. (2014). Evidence for Inflammation-Induced Bone Loss in Non-Radiographic Axial Spondyloarthritis. Rheumatology.

[109] Rossini M., Viapiana O., Idolazzi L., Ghellere F., Fracassi E., Troplini S., Povino M.R., Kunnathully V., Adami S., Gatti D. (2016). Higher Level of Dickkopf-1 is Associated with Low Bone Mineral Density and Higher Prevalence of Vertebral Fractures in Patients with Ankylosing Spondylitis. Calcif. Tissue Int..

[110] Maillefert J.F., Aho L.S., El Maghraoui A., Dougados M., Roux C. (2001). Changes in Bone Density in Patients with Ankylosing Spondylitis: A Two-Year Follow-Up Study. Osteoporos. Int..

[111] Wang Y.F., Teng M.M.H., Chang C.Y., Wu H.T., Wang S.T. (2005). Imaging Manifestations of Spinal Fractures in Ankylosing Spondylitis. AJNR Am. J. Neuroradiol..

[112] Geusens P., Vosse D., Van Der Linden S. (2007). Osteoporosis and Vertebral Fractures in Ankylosing Spondylitis. Curr. Opin. Rheumatol..

[113] Montala N., Juanola X., Collantes E., Munoz-Gomariz E., Gonzalez C., Gratacos J., Zarco P., Fernandez Sueiro J.L., Mulero J., Torre-Alonso J.C. (2011). Prevalence of Vertebral Fractures by Semiautomated Morphometry in Patients with Ankylosing Spondylitis. J. Rheumatol..

[114] Vosse D., Landewe R., Van Der Heijde D., Van Der Linden S., Van Staa T.P., Geusens P. (2009). Ankylosing Spondylitis and the Risk of Fracture: Results from a Large Primary Care-Based Nested Case-Control Study. Ann. Rheum. Dis..

[115] Beek K.J., Rusman T., Van Der Weijden M.A.C., Lems W.F., Van Denderen J.C., Konsta M., Visman I., Nurmohamed M.T., Van Der Horst-Bruinsma I.E. (2019). Long-Term Treatment with TNF-Alpha Inhibitors Improves Bone Mineral Density But Not Vertebral Fracture Progression in Ankylosing Spondylitis. J. Bone Miner. Res..

[116] Ramirez J., Nieto-Gonzalez J.C., Curbelo Rodriguez R., Castaneda S., Carmona L. (2018). Prevalence and Risk Factors for Osteoporosis and Fractures in Axial Spondyloarthritis: A Systematic Review and Meta-Analysis. Semin. Arthritis Rheum..

[117] Ogdie A., Harter L., Shin D., Baker J., Takeshita J., Choi H.K., Love T.J., Gelfand J.M. (2017). The Risk of Fracture Among Patients with Psoriatic Arthritis and Psoriasis: A Population-Based Study. Ann. Rheum. Dis..

[118] Neumann A., Haschka J., Kleyer A., Schuster L., Englbrecht M., Berlin A., Figueiredo C.P., Simon D., Muschitz C., Kocijan R. (2018). Cortical Bone Loss is An Early Feature of Nonradiographic Axial Spondyloarthritis. Arthritis Res. Ther..

[119] Gamsjaeger S., Srivastava A.K., Wergedal J.E., Zwerina J., Klaushofer K., Paschalis E.P., Tatakis D.N. (2014). Altered Bone Material Properties in HLA-B27 Rats Include Reduced Mineral to Matrix Ratio and Altered Collagen Cross-Links. J. Bone Miner. Res..

[120] Rauner M., Thiele S., Fert I., Araujo L.M., Layh-Schmitt G., Colbert R.A., Hofbauer C., Bernhardt R., Burki A., Schwiedrzik J. (2015). Loss of Bone Strength in HLA-B27 Transgenic Rats is Characterized by a High Bone Turnover and is Mainly Osteoclast-Driven. Bone.

[121] Stupphann D., Rauner M., Krenbek D., Patsch J., Pirker T., Muschitz C., Resch H., Pietschmann P. (2008). Intracellular and Surface RANKL are Differentially Regulated in Patients with Ankylosing Spondylitis. Rheumatol. Int..

[122] Hauser B., Zhao S., Visconti M.R., Riches P.L., Fraser W.D., Piec I., Goodson N.J., Ralston S.H. (2017). Autoantibodies to Osteoprotegerin are Associated with Low Hip Bone Mineral Density and History of Fractures in Axial Spondyloarthritis: A Cross-Sectional Observational Study. Calcif. Tissue Int..

[123] Bernstein C.N., Benchimol E.I., Bitton A., Murthy S.K., Nguyen G.C., Lee K., Cooke-Lauder J., Kaplan G.G. (2019). The Impact of Inflammatory Bowel Disease in Canada 2018: Extra-intestinal Diseases in IBD. J. Can. Assoc. Gastroenterol..

[124] Viapiana O., Gatti D., Idolazzi L., Fracassi E., Adami S., Troplini S., Povino M.R., Rossini M. (2014). Bisphosphonates Vs Infliximab in Ankylosing Spondylitis Treatment. Rheumatology.

[125] Mok C.C., Li O.C., Chan K.L., Ho L.Y., Hui P.K. (2015). Effect of Golimumab and Pamidronate on Clinical Efficacy and MRI Inflammation in Axial Spondyloarthritis: A 48-Week Open Randomized Trial. Scand. J. Rheumatol..

[126] Munoz-Ortego J., Vestergaard P., Rubio J.B., Wordsworth P., Judge A., Javaid M.K., Arden N.K., Cooper C., Diez-Perez A., Prieto-Alhambra D. (2014). Ankylosing Spondylitis is Associated with an Increased Risk of Vertebral and Nonvertebral Clinical Fractures: A Population-Based Cohort Study. J. Bone Miner. Res..

[127] Haroon N.N., Sriganthan J., Al Ghanim N., Inman R.D., Cheung A.M. (2014). Effect of TNF-Alpha Inhibitor Treatment on Bone Mineral Density in Patients with Ankylosing Spondylitis: A Systematic Review and Meta-Analysis. Semin. Arthritis Rheum..

[128] Barnabe C., Hanley D.A. (2009). Effect of Tumor Necrosis Factor Alpha Inhibition on Bone Density and Turnover Markers in Patients with Rheumatoid Arthritis and Spondyloarthropathy. Semin. Arthritis Rheum..

[129] Simon D., Kleyer A., Bayat S., Tascilar K., Kampylafka E., Meinderink T., Schuster L., Petrov R., Liphardt A.M., Rech J. (2019). Effect of Disease-Modifying anti-Rheumatic Drugs on Bone Structure and Strength in Psoriatic Arthritis Patients. Arthritis Res. Ther..

[130] Tsokos G.C. (2011). Systemic Lupus Erythematosus. N. Engl. J. Med..

[131] Bultink I.E.M., Lems W.F. (2016). Lupus and Fractures. Curr. Opin. Rheumatol..

[132] Mahmoud K., Zayat A., Vital E.M. (2017). Musculoskeletal Manifestations of Systemic Lupus Erythmatosus. Curr. Opin. Rheumatol..

[133] Fanouriakis A., Kostopoulou M., Alunno A., Aringer M., Bajema I., Boletis J.N., Cervera R., Doria A., Gordon C., Govoni M. (2019). 2019 update of the EULAR recommendations for the management of systemic lupus erythematosus. Ann. Rheum. Dis..

[134] Orsolini G., Bultink I.E.M., Adami G., Fassio A., Viapiana O., Giollo A., Gatti D., Rossini M. (2019). Bone health, an Often Forgotten Comorbidity in Systemic Lupus Erythematosus: A Comment on the New Recomendationms. Ann. Rheum. Dis..

[135] Tedeschi S.K., Kim S.C., Guan H., Grossman J.M., Costenbader K.H. (2019). Comparative Fracture Risks Among United States Medicaid Enrollees with and Those without Systemic Lupus Erythematosus. Arthritis Rheumatol..

[136] Rees F., Doherty M., Grainge M.J., Lanyon P., Zhang W. (2017). The Worldwide Incidence and Prevalence of Systemic Lupus Erythematosus: A Systematic Review of Epidemiological Studies. Rheumatology.

[137] Ralston S.H., Schett G. (2018). Osteoimmunology. Calcif. Tissue Int..

[138] Rossini M., Bagnato G., Frediani B., Iagnocco A., LA Montagna G., Minisola G., Caminiti M., Varenna M., Adami S. (2011). Relationship of Focal Erosions, Bone Mineral Density, and Parathyroid Hormone in Rheumatoid Arthritis. J. Rheumatol..

[139] Bonfa A.C., Seguro L.P.C., Caparbo V., Bonfa E., Pereira R.M.R. (2015). RANKL and OPG Gene Polymorphisms: Associations with Vertebral Fractures and Bone Mineral Density in Premenopausal Systemic Lupus Erythematosus. Osteoporos. Int..

[140] Ceccarelli F., Perricone C., Colasanti T., Massaro L., Cipriano E., Pendolino M., Natalucci F., Mancini R., Spinelli F.R., Valesini G. (2018). Anti-Carbamylated Protein Antibodies as a New Biomarker of Erosive Joint Damage in Systemic Lupus Erythematosus. Arthritis Res. Ther..

[141] Regueiro C., Ortiz A.M., Boveda M.D., Castaneda S., Gonzalez-Alvaro I., Gonzalez A. (2018). Association of High Titers of Anti-Carbamylated Protein Antibodies with Decreased Bone Mineral Density in Early Arthritis Patients. PLoS ONE.

[142] Kuhn A., Gensch K., Haust M., Meuth A.M., Boyer F., Dupuy P., Lehmann P., Metze D., Ruzicka T. (2011). Photoprotective Effects of a Broad-Spectrum Sunscreen in Ultraviolet-Induced Cutaneous Lupus Erythematosus: A Randomized, Vehicle-Controlled, Double-Blind Study. J. Am. Acad. Dermatol..

[143] Lim S.H.L., Benseler S.M., Tyrrell P.N., Charron M., Harvey E., Hebert D., Silverman E.D. (2011). Low Bone Mineral Density is Present in Newly Diagnosed Paediatric Systemic Lupus Erythematosus Patients. Ann. Rheum. Dis..

[144] Caetano M., Terreri M.T., Ortiz T., Pinheiro M., Souza F., Sarni R. (2015). Bone Mineral Density Reduction in Adolescents with Systemic Erythematosus Lupus: Association with Lack of Vitamin D Supplementation. Clin. Rheumatol..

[145] Almehed K., Forsblad D’Elia H., Kvist G., Ohlsson C., Carlsten H. (2007). Prevalence and Risk Factors of Osteoporosis in Female SLE Patients-Extended Report. Rheumatology.

[146] Wang X., Yan S., Liu C., Xu Y., Wan L., Wang Y., Gao W., Meng S., Liu Y., Liu R. (2016). Fracture risk and Bone Mineral Density Levels in Patients with Systemic Lupus Erythematosus: A Systematic Review and Meta-Analysis. Osteoporos. Int..

[147] Jacobs J., Sjoe M.W.P.T.A., Voskuyl A.E., Lems W.F., Bultink I.E.M. (2015). Unexpected Severe Incident Vertebral Fractures in Patients with Systemic Lupus Erythematosus: Comment on the Article By Zhu et al. Lupus.

[148] Lee D., Kim H., Ahn S.H., Lee S.H., Bae S.J., Kim E.H., Kim H.K., Choe J.W., Kim B.J., Koh J.M. (2015). The Association Between Serum Dehydroepiandrosterone Sulphate (DHEA-S) Level and Bone Mineral Density in Korean Men. Clin. Endocrinol..

[149] Lahita R.G., Bradlow H.L., Ginzler E., Pang S., New M. (1987). Low Plasma Androgens in Women with Systemic Lupus Erythematosus. Arthritis Rheum..

[150] Formiga F., Moga I., Nolla J.M., Navarro M.A., Bonnin R., Roig-Escofet D. (1997). The Association of Dehydroepiandrosterone Sulphate Levels with Bone Mineral Density in Systemic Lupus Erythematosus. Clin. Exp. Rheumatol..

[151] Tuin J., Sanders J.S.F., Van Beek A.P., Hoek A., Stegeman C.A. (2016). Brief Report: Menopause and Primary Ovarian Insufficiency in Women Treated for Antineutrophil Cytoplasmic Antibody-Associated Vasculitides. Arthritis Rheumatol..

[152] Orsolini G., Viapiana O., Rossini M., Bonifacio M., Zanotti R. (2018). Bone Disease in Mastocytosis. Immunol. Allergy Clin. N. Am..

[153] Sarkissian A., Sivaraman V., Bout-Tabaku S., Ardoin S.P., Moore-Clingenpeel M., Mruk V., Steigelman H., Morris K., Bowden S.A. (2019). Bone Turnover Markers in Relation to Vitamin D Status and Disease Activity in Adults with Systemic Lupus Erythematosus. Lupus.

[154] Stagi S., Cavalli L., Bertini F., Matucci Cerinic M., Luisa Brandi M., Falcini F. (2014). Cross-sectional and Longitudinal Evaluation of Ass and quality in Children and Young Adults with Juvenile Onset Systemic Lupus Erythematosus (JSLE): Role of Bone Mass Determinants Analyzed by DXA, PQCT and QUS. Lupus.

[155] Paupitz J.A., Lima G.L., Alvarenga J.C., Oliveira R.M., Bonfa E., Pereira R.M.R. (2016). Bone Impairment Assessed by HR-pQCT in Juvenile-Onset Systemic Lupus Erythematosus. Osteoporos. Int..

[156] Bultink I.E.M., Lems W.F., Kostense P.J., Dijkmans B.A.C., Voskuyl A.E. (2005). Prevalence of and Risk Factors for Low Bone Mineral Density and Vertebral Fractures in Patients with Systemic Lupus Erythematosus. Arthritis Rheum..

[157] Lakshminarayanan S., Walsh S., Mohanraj M., Rothfield N. (2001). Factors Associated with Low Bone Mineral Density in Female Patients with Systemic Lupus Erythematosus. J. Rheumatol..

[158] Cramarossa G., Urowitz M.B., Su J., Gladman D., Touma Z. (2017). Prevalence and Associated Factors of Low Bone Mass in Adults with Systemic Lupus Erythematosus. Lupus.

[159] Zhu T.Y., Griffith J.F., Au S.K., Tang X.L., Kwok A.W., Leung P.C., Li E.K., Tam L.S. (2014). Incidence of and Risk Factors for Non-Vertebral and Vertebral Fracture in FEMALE CHINESE Patients with Systemic Lupus Erythematosus: A Five-Year Cohort Study. Lupus.

[160] Mendoza-Pinto C., Rojas-Villarraga A., Molano-Gonzalez N., Jimenez-Herrera E.A., Leon-Vazquez M., De La L., Montiel-Jarquin A., Garcia-Carrasco M., Cervera R. (2018). Bone Mineral Density and Vertebral Fractures in Patients with Systemic Lupus Erythematosus: A Systematic Review and Meta-Regression. PLoS ONE.

[161] Mok C.C., Mak A., Ma K.M. (2005). Bone Mineral Density in Postmenopausal Chinese Patients with Systemic Lupus Erythematosus. Lupus.

[162] Wang S.H., Chang Y.S., Liu C.J., Lai C.C., Chen W.S., Chen T.J., Wang S.J. (2013). Association of Systemic Lupus Erythematosus with A Higher Risk of Cervical But Not Trochanteric Hip Fracture: A Nationwide Population-Based Study. Arthritis Care Res..

[163] Furnrohr B.G., Rhodes B., Munoz L.E., Weiis K., Vyse T.J., Schett G. (2015). Osteoclast Differentiation Is Impaired in a Subgroup of SLE Patients and Correlates Inversely with Mycophenolate Mofetil Treatment. Int. J. Mol. Sci..

[164] Omair M.A., Pagnoux C., McDonald-Blumer H., Johnson S.R. (2013). Low Bone Density in Systemic Sclerosis. A Systematic Review. J. Rheumatol..

[165] La Montagna G., Baruffo A., Abbadessa S., Maja L., Tirri R. (1995). Evidence for Bone Resorption in Systemic Sclerosis. J. Rheumatol..

[166] La Montagna G., Vatti M., Valentini G., Tirri G. (1991). Osteopenia in systemic sclerosis. Evidence of a participating role of earlier menopause. Clin. Rheumatol..

[167] Loucks J., Pope J.E. (2005). Osteoporosis in Scleroderma. Semin Arthritis Rheum..

[168] Frediani B., Baldi F., Falsetti P., Acciai C., Filippou G., Spreafico A., Siagri C., Chellini F., Capperucci C., Filipponi P. (2004). Clinical Determinants of Bone Mass and Bone Ultrasonometry in Patients with Systemic Sclerosis. Clin. Exp. Rheumatol..

[169] Frediani B., Baldi F., Falsetti P., Acciai C., Filippou G., Spreafico A., Chellini F., Capperucci C., Filipponi P., Galeazzi M. (2004). Bone Mineral Density in Patients with Systemic Sclerosis. Ann. Rheum. Dis..

[170] Carbone L., Tylavsky F., Wan J., McKown K., Cheng S. (1999). Bone Mineral Density in Scleroderma. Rheumatology.

[171] Di Munno O., Mazzantini M., Massei P., Ferdeghini M., Pitaro N., Latorraca A., Ferri C. (1995). Reduced Bone Mass and Normal Calcium Metabolism in Systemic Sclerosis with and without Calcinosis. Clin. Rheumatol..

[172] Sampaio-Barros P.D., Costa-Paiva L., Filardi S., Sachetto Z., Samara A.M., Marques-Neto J.F. (2005). Prognostic Factors of Low Bone Mineral Density in Systemic Sclerosis. Clin. Exp. Rheumatol..

[173] Souza R.B., Borges C.T., Takayama L., Aldrighi J.M., Pereira R.M. (2006). Systemic Sclerosis and Bone Loss: The Role of the Disease and Body Composition. Scand. J. Rheumatol..

[174] Caimmi C., Caramaschi P., Barausse G., Orsolini G., Idolazzi L., Gatti D., Viapiana O., Adami S., Biasi D., Rossini M. (2016). Bone Metabolism in a Large Cohort of Patients with Systemic Sclerosis. Calcif. Tissue Int..

[175] Antonelli A., Ferri C., Fallahi P., Cazzato M., Ferrari S.M., Sebastiani M., Ferrannini E. (2007). Clinical and Subclinical Autoimmune Thyroid Disorders in Systemic Sclerosis. Eur. J. Endocrinol..

[176] Kucharz E.J. (1993). Thyroid Disorders in Patients with Progressive Systemic Sclerosis: A Review. Clin. Rheumatol..

[177] Vacca A., Cormier C., Piras M., Mathieu A., Kahan A., Allanore Y., Vitamin D. (2009). Deficiency and Insufficiency in 2 Independent Cohorts of Patients with Systemic Sclerosis. J. Rheumatol..

[178] Gambichler T., Chrobok I., Hoxtermann S., Kreuter A. (2011). Significantly Decreased Serum 25-Hydroxyvitamin d Levels in a Large German Systemic Sclerosis Cohort. J. Rheumatol..

[179] Caramaschi P., Dalla Gassa A., Ruzzenente O., Volpe A., Ravagnani V., Tinazzi I., Barausse G., Bambara L.M., Biasi D. (2010). Very Low Levels of Vitamin D in Systemic Sclerosis Patients. Clin. Rheumatol..

[180] Caimmi C., Bertoldo E., Pozza A., Caramaschi P., Orsolini G., Gatti D., Rossini M., Viapiana O. (2019). Vitamin D Serum Levels and the Risk of Digital Ulcers in Systemic Sclerosis: A Longitudinal Study. Int. J. Rheum. Dis..

[181] Trombetta A.C., Smith V., Gotelli E., Ghio M., Paolino S., Pizzorni C., Vanhaecke A., Ruaro B., Sulli A., Cutolo M. (2017). Vitamin D Deficiency and Clinical Correlations in Systemic Sclerosis Patients: A Retrospective Analysis for Possible Future Developments. PLoS ONE.

[182] Piersma B., Bank R.A., Boersema M. (2015). Signaling in Fibrosis: TGF-β, WNT, and YAP/TAZ Converge. Front. Med..

[183] Tamanini S., Idolazzi L., Gatti D., Viapiana O., Fassio A., Rossini M. (2013). Insight into the WNT System and its Drug Related Response. Reumatismo.

[184] Ruaro B., Casabella A., Paolino S., Pizzorni C., Ghio1 M., Seriolo C., Molfetta L., Odetti P., Smith V. (2018). Cutolo M Dickkopf-1 (Dkk-1) Serum Levels in Systemic Sclerosis and Rheumatoid Arthritis Patients: Correlation with the Trabecular Bone Score (TBS). Clinical. Rheumatol..

[185] Taylan A., Birlik M., Kenar G., Toprak B., Gundogdu B., Gurler O., Karakas B., Akinci B., Sisman A.R. (2019). Osteoprotegrin Interacts with Biomarkers and Cytokines that Have Roles in Osteoporosis, Skin Fibrosis, and Vasculopathy in Systemic Sclerosis: A Potential Multifaceted Relationship Between OPG/ RANKL/TRAIL and Wnt Inhibitors. Mod. Rheumatol..

[186] Tu X., Rhee Y., Condon K.W., Bivi N., Allen M.R., Dwyer D., Stolina M., Turner C.H., Robling A.G., Plotkin L.I. (2012). Sost Downregulation and Local Wnt Signaling are Required for the Osteogenic Response to Mechanical Loading. Bone.

[187] Dovio A., Data V., Carignola R., Calzolari G., Vitetta R., Ventura M., Saba L., Severino A., Angeli A. (2008). Circulating Osteoprotegerin and Soluble RANK Ligand in Systemic Sclerosis. J. Rheumatol..

[188] Forde H., Harper E., Davenport C., Rochfort K.D., Wallace R., Murphy R.P., Smith D., Cummins P.M. (2016). The Beneficial Pleiotropic Effects of Tumour Necrosis Factor-Related Apoptosis-Inducing Ligand (TRAIL) within the Vasculature: A Review of the Prova. Atherosclerosis.

[189] Koumakis E., Avouac J., Winzenrieth R., Toth E., Payet J., Kahan A., Allanore Y. (2015). Cormier C Trabecular Bone Score in Female Patients with Systemic Sclerosis: Comparison with Rheumatoid Arthritis and Influence of Glucocorticoid Exposure. J. Rheumatol..

[190] Fauny M., Bauer E., Albuisson E., Perrier-Cornet J., Deibener J., Chabot F., Mandry D., Huttin O., Chary-Valckenaere I., Loeuille D. (2018). Vertebral Fracture Prevalence and Measurement of the Scanographic Bone Attenuation Coefficient on CT-Scan in Patients with Systemic Sclerosis. Rheumatol. Int..

[191] Atteritano M., Sorbara S., Bagnato G., Miceli G., Sangari D., Morgante S., Visalli E., Bagnato G. (2013). Bone Mineral Density, Bone Turnover Markers and Fractures in Patients with Systemic Sclerosis: A Case Control Study. PLoS One.

[192] Avouac J., Koumakis E., Toth E., Meunier M., Maury E., Kahan A., Cormier C., Allanore Y. (2012). Increased Risk of Osteoporosis and Fracture in Women with Systemic Sclerosis: A Comparative Study with Rheumatoid Arthritis. Arthritis Care Res..

[193] Kermani T.A., Sreih A.G., Cuthbertson D., Carette S., Hoffman G.S., Khalidi N.A., Koening C.L., Langford C.A., McAlear C.A., Monach P.A. (2018). Evaluation of Damage in Giant Cell Arteritis. Rheumatol. Int. Rheumatol..

[194] Dasgupta B., Borg F.A., Hassan N., Alexander L., Barraclough K., Bourke B., Fulcher J., Hollywood J., Hutchings A., James P. (2010). BSR and BHPR Guidelines for the Management of Giant Cell Arteritis. Rheumatology.

[195] Paskins Z., Whittle R., Sultan A.A., Muller S., Blagojevic-Bucknall M., Helliwell T., Hider S., Roddy E., Mallen C. (2018). Risk of Fracture Among Patients with Polymyalgia Rheumatica and Giant Cell Arteritis: A Population-Based Study. BMC Medicine..

[196] Englund M., Merkel P.A., Tomasson G., Segelmark M., Mohammad A.J. (2016). Comorbidities in Patients with Antineutrophil Cytoplasmic Antibody-Associated Vasculitis Versus the General Population. J. Rheumatol..

[197] Boomsma M.M., Stegeman C.A., Kramer A.B., Karsijns M., Piers D.A., Tervaert J.W. (2002). Prevalence of Reduced Bone Mineral Density in Patients with Anti-Neutrophil Cytoplasmic Antibody Associated Vasculitis and the Role of Immunosuppressive Therapy: A Cross-Sectional Study. Osteoporos. Int. A J. Establ. Result Coop. Eur. Found. Osteoporos. Natl. Osteoporos. Found. USA.

[198] Samson M., Puechal X., Devilliers H., Ribi C., Cohen P., Bienvenu B., Ruivard M., Terrier B., Pagnoux C., Mouthon L. (2014). Long-Term Follow-Up of a Randomized Trial on 118 Patients with Polyarteritis Nodosa Or Microscopic Polyangiitis without Poor-Prognosis Factors. Autoimmun. Rev..

[199] Robson J., Doll H., Suppiah R., Flossmann O., Harper L., Hoglund P., Jayne D., Mahr A., Westman K., Luqmani R. (2015). Damage in the Anca-Associated Vasculitides: Long-Term Data from the European Vasculitis Study Group (EUVAS) Therapeutic Trials. Ann. Rheum. Dis..

[200] Hu Z., Zhu S., Zhang B. (2016). Impaired Bone Health in Patients with Primary Sjogren’s Syndrome. Arthritis Rheumatol..

[201] Lai E.L., Huang W.N. (2019). Ten-Year Fracture Risk by FRAX and Osteoporotic Fractures in Patients with Systemic Autoimmune Diseases. Lupus.

[202] Kleiber Balderrama C., Rosenthal A.K., Lans D., Singh J.A., Bartels C.M. (2017). Calcium Pyrophosphate Deposition Disease and Associated Medical Comorbidities: A National Cross-Sectional Study of US Veterans. Arthritis Care Res..

[203] Makovey J., Macara M., Chen J.S., Hayward C.S., March L., Seibel M.J., Sambrook P.N. (2013). Serum Uric Acid Plays a Protective Role for Bone Loss in Peri-And Postmenopausal Women: A Longitudinal Study. Bone.

[204] Muka T., De Jonge E.A., Kiefte-De Jong J.C., Uitterlinden A.G., Hofman A., Dehghan A., Zillikens M.C., Franco O.H., Rivadeneira F. (2016). The Influence of Serum Uric Acid on Bone Mineral Density, Hip Geometry, and Fracture Risk: The Rotterdam Study. J. Clin. Endocrinol. Metab..

[205] Lin X., Zhao C., Qin A., Hong D., Liu W., Huang K., Mo J., Yu H., Wu S., Fan S. (2015). Association Between Serum Uric Acid and Bone Health in General Population: A Large and Multicentre Study. Oncotarget.

[206] Kim B.J., Baek S., Ahn S.H., Kim S.H., Jo M.W., Bae S.J., Kim H.K., Choe J., Park G.M., Kim Y.H. (2014). Higher Serum Uric Acid as a Protective Factor Against Incident Osteoporotic Fractures in Korean Men: A Longitudinal Study Using the National Claim Registry. Osteoporos. Int..

[207] Dalbeth N., Topless R., Flynn T., Cadzow M., Bolland M.J., Merriman T.R. (2015). Mendelian Randomization Analysis to Examine for a Causal Effect of Urate on Bone Mineral Density. J. Bone Miner. Res..

[208] Xiong A., Yao Q., He J., Fu W., Yu J., Zhang Z. (2016). No Causal Effect of Serum Urate on Bone-Related Outcomes Among a Population of Postmenopausal Women and Elderly Men of Chinese Han Ethnicity--a Mendelian Randomization Study. Osteoporos. Int..

[209] Kok V.C., Horng J.T., Wang M.N., Chen Z.Y., Kuo J.T., Hung G.D. (2018). Gout as a Risk Factor for Osteoporosis: Epidemiologic Evidence from a Population-Based Longitudinal Study Involving 108,060 Individuals. Osteoporos. Int. A J. Establ. Result Coop. Eur. Found. Osteoporos. Natl. Osteoporos. Found. USA.

[210] Kim S.C., Paik J.M., Liu J., Curhan G.C., Solomon D.H. (2017). Gout and the Risk of Non-Vertebral Fracture. J. Bone Miner. Res. Off. J. Am. Soc. Bone Mineral. Res..

